# 

**Published:** 1887-04

**Authors:** 


					﻿Note : The word cases at the head of the
article on page 136 (plural) is a misprint. It
should be case. The error was discovered only
after the sheets had passed through the press,
too late to correct.—Eds.
NOTICE.
All articles for publication, books for review, business communications, etc.,
must be sent to Editors, 1123 Spruce Street, Philadelphia.
Subscribers will please notice that this Journal will be sent until arrears
are paid and it is ordered discontinued.
tesY* Subscribers, Contributors, and Exchanges,
please TAKE NOTICE. The Office of THIS
JOURNAL has been removed to 1123 Spruce
Street, where all communications must be sent.
All subscriptions are due in advance; subscribers will therefore
please forward their subscriptions at once; let each subscriber see
that his account is settled promptly.
Subscribers are respectf ully requested to make all checks
and money-orders payable to THE HOMCEOPATIIIC
*PHYSICIAN, and not to either of the Editors.
				

